# Dosis de radiación por radiografías y factores asociados en neonatos de la Unidad de Recién Nacidos del Hospital Universitario San Ignacio, Bogotá, Colombia

**DOI:** 10.7705/biomedica.6668

**Published:** 2023-09-30

**Authors:** Diana Ramírez, Víctor Ramos, Sandra Navarro, Adriana Montealegre, Julia Arciniegas

**Affiliations:** 1 Departamento de Pediatría, Hospital Universitario San Ignacio, Bogotá, D.C., Colombia Departamento de Pediatría Hospital Universitario San Ignacio Bogotá, D.C. Colombia; 2 Facultad de Medicina, Pontificia Universidad Javeriana, Bogotá, D.C., Colombia Pontificia Universidad Javeriana Facultad de Medicina Pontificia Universidad Javeriana Bogotá, D.C. Colombia; 3 Departamento de Radiología e Imágenes Diagnósticas, Hospital Universitario San Ignacio, Bogotá, D.C., Colombia Departamento de Radiología e Imágenes Diagnósticas Hospital Universitario San Ignacio Bogotá, D.C. Colombia

**Keywords:** recién nacido, dosis de radiación, radiación, radiografías, factores de riesgo, Infant, newborn, radiation dosage, radiation, radiography, risk factors

## Abstract

**Introducción.:**

Las radiografías continúan usándose ampliamente, subestimando los riesgos. Esto sucede, especialmente, en las unidades de cuidado neonatal, lo que implica que los neonatos reciben una dosis de radiación ionizante mayor que los adultos.

**Objetivo.:**

Cuantificar las dosis de radiación recibidas al tomar radiografías y evaluar los posibles factores asociados con el aumento de la dosis.

**Materiales y métodos.:**

Se llevó a cabo un estudio observacional de 160 neonatos de la Unidad de Recién Nacidos del Hospital Universitario San Ignacio, Bogotá, Colombia. Se consideró como variable dependiente la dosis de entrada en piel por cada radiografía. Se hizo la caracterización de los pacientes, seguida de un análisis multivariado con regresión lineal múltiple para identificar factores asociados.

**Resultados.:**

Se analizaron 160 pacientes y 492 radiografías en total. Entre los hallazgos más frecuentes, se encuentran: pacientes de sexo masculino (n=87; 54,4 %), nacimiento por cesárea (n=122; 76,3 %) e indicación de toma de radiografías por dificultad respiratoria (n=123; 24,9 %). El 1,8 % (n=9) de los pacientes no tenían una indicación para la toma de la radiografía. La radiografía más frecuente fue la de tórax (n=322; 65,4 %). La mayoría de las radiografías se tomaron con el equipo computarizado (n=352; 71,5 %) y no con el digital (n=140, 28,4 %). La mediana de la dosis de entrada en piel con el equipo computarizado fue de 0,112 mGy (0,022; 0,134 mGy) y, con el equipo digital, de 0,020 mGy (0,019, 0,022 mGy).

**Conclusiones.:**

Se cuantificaron las dosis de radiación absorbida en neonatos, general y específica, con el equipo computarizado y el digital. Se identificaron mayores dosis con el equipo computarizado. Se reconoció la interacción entre el equipo computarizado con menores edades gestacionales corregidas como principal factor para el aumento de la dosis. Además, se reconoció la relación entre el equipo computarizado y una menor edad gestacional corregida, como principal factor para una mayor dosis.

## Abstract (en)

**Introduction.:**

Radiographs are still widely used, underestimating the risks. This situation is frequent in neonatal care units, generating radiation doses than in adults.

**Objective.:**

To quantify the received radiation doses when performing radiographs on neonates and the possible factors associated with higher doses.

**Materials and methods.:**

We performed an observational study of 160 neonates from the newborn unit of the *Hospital Universitario San Ignacio*, Bogotá, Colombia. We considered the input dose of each radiograph as the dependent variable. Patients were characterized and a multivariate analysis with multiple linear regression was performed to identify associated factors.

**Results.:**

We analyzed 160 newborns and 492 radiographs. The most frequent findings were male patients (n=87, 54.4%), cesarean delivery (n=122, 76.3%), and radiograph indication for respiratory distress (n=123, 24.9%). One-point eight percent of the patients (n=9) did not have radiograph indication. The most frequently taken radiograph was chest (322, 65.4%). Most radiographs were taken with a computerized equipment (n=352, 71.5%), compared to a digital one (n=140, 28.4%). The median input dose with computerized equipment was 0.112 mGy (0.022, 0.134 mGy), and with the digital equipment was 0.020 mGy (0.019, 0.022 mGy).

**Conclusions.:**

The general and specific absorbed radiation doses were measured in neonates with a computerized and a digital equipment. We identified higher doses with the computerized equipment. In addition, it was recognized the correlation between computerized radiography equipment with lower corrected gestational ages as the main factor for dose increase.

En las últimas décadas, la radiología diagnóstica ha venido en constante transformación y modifica día a día la práctica clínica con resultados sorprendentes. En la actualidad, se cuenta con estudios imagenológicos y, en ocasiones, se subestiman los riesgos que conlleva. En las unidades de cuidado neonatal, la edad de los pacientes es un factor crítico frente a la exposición a la radiación, por tres razones principales. La primera es que, durante el desarrollo fetal, se produce gran proliferación y diferenciación de los tejidos, y se sabe que estas células son más propensas a desarrollar cáncer inducido por radiación. La segunda es que se presume que este grupo etario tiene una esperanza de vida más larga. La tercera es que el tamaño corporal de los recién nacidos (especialmente, los prematuros) es pequeño, por lo cual el haz primario de radiación impacta sobre la gran mayoría de los órganos, lo que se traduce en una mayor dosis acumulativa ^1^^-^^4^.

Organizaciones internacionales, como la Comisión Internacional de Protección Radiológica y el Organismo Internacional de Energía Atómica, han establecido como un requisito fundamental, la optimización de la protección radiológica de los pacientes en el uso médico de las radiografías. En Estados Unidos, por ejemplo, la *U.S. Food and Drug Administration* lanzó una campaña para reducir la radiación en niños, creando estrategias para disminuir la cantidad de exposición de radiación en esta población. Este tipo de estrategias no existen en Colombia. Todas y cada una de estas organizaciones, leyes y resoluciones, apuntan a que cada institución debe contar con estimaciones de riesgo de acuerdo con los equipos disponibles, los métodos de medición, las técnicas, etc. ^5^^,^^6^.

En la radiografía convencional, la exposición de los pacientes de forma directa al haz de radiación se caracteriza, generalmente, mediante dos parámetros: la dosis de entrada en piel y el producto dosis-área. Sin embargo, aunque estas magnitudes no representan la dosis absorbida por el paciente, se recomienda su uso para establecer directrices o protocolos institucionales útiles para optimizar la protección contra la exposición radiológica de los pacientes. Estos esfuerzos de optimización deben priorizarse en función del riesgo potencial de efectos estocásticos para los pacientes, como aquellos que resultan por dosis elevadas en órganos o los que implican exposición de órganos más radiosensibles ^7^.

La estimación de la dosis de radiación en los neonatos de la población colombiana permite identificar riesgos y garantizar el uso de dosis óptimas para lograr el diagnóstico mediante radiografías. Esto pretende generar conciencia en el personal de salud acerca de las indicaciones de la toma de las radiografías y reducir el número de radiografías repetidas. Además, los valores de estas dosis pueden utilizarse como referencias, con el fin de comparar las dosis que reciben los pacientes en otras instituciones de salud, lo cual favorece la optimización de la técnica radiológica, aplicando medidas correctivas en procedimientos cuyas dosis sean superiores a las de referencia.

Por esto, el objetivo de este estudio fue cuantificar las dosis de radiación recibidas durante los estudios radiográficos y determinar los posibles factores de riesgo asociados en los neonatos de la Unidad de Recién Nacidos del Hospital Universitario San Ignacio, Bogotá, Colombia, teniendo en cuenta la exposición a la radiación directa y a la dispersa. 

## Materiales y métodos

La población de estudio corresponde a neonatos hospitalizados en la Unidad de Recién Nacidos del Hospital Universitario San Ignacio, Bogotá, Colombia, entre abril y septiembre de 2021. Se excluyeron aquellos pacientes cuyos padres no desearon participar. Se determinó un tamaño de muestra de 160 pacientes. En primer lugar, los tecnólogos en radiología diligenciaron antes de la toma: número de historia clínica, miliamperios (mAs), kilovoltaje (kV), distancia del punto focal al paciente, espesor del paciente (medida anteroposterior en centímetros de cada cuerpo) y tipo de radiografía. Luego, se revisaron las historias clínicas para identificar variables clínicas y sociodemográficas, como: edad cronológica y corregida al momento de la toma de la radiografía, sexo, edad materna, peso y talla del día de la toma de la radiografía, vía de parto y enfermedades o condiciones relacionadas con la indicación del estudio. La información se registró en una base de datos REDcap (*Research Electronic Data capture*).

Las radiografías fueron tomadas con dos equipos de rayos X portátiles, uno con tecnología de radiografía computarizada y otro con radiografía digital directa.

Con los datos obtenidos, se cuantificó la dosis de entrada en piel, mediante la curva de rendimiento a 1 metro, en función del kilovoltaje de cada uno de los equipos portátiles de rayos X. La curva se obtuvo con medidas de Kerma en aire realizadas con una cámara de ionización de 3 ml. El Kerma de entrada en el paciente se determinó reemplazando el kilovoltaje usado en la radiografía en la curva de rendimiento, corrigiendo este valor según la distancia de foco a superficie y multiplicando por el miliamperaje. Finalmente, para calcular la dosis de entrada en la superficie, se multiplicó el Kerma por un factor de retrodispersión que correspondió al promedio de los factores de retrodispersión calculados con base en las mediciones realizadas de Kerma en aire y Kerma con retrodispersión. Esto se hizo sobre láminas de polimetilmetacrilato de 5, 6 y 7 cm de espesor, para kV de 57, 60 y 63 y mAs de 0,25, 0,5, 1, 1,2 y 1,6, con la cámara de ionización de 3 ml.

Se usó el *software* CALDose X, que utiliza el código Monte Carlo EGScnr para estimar las dosis recibidas por órganos y tejidos, y así, calcular la dosis efectiva teniendo en cuenta los factores de ponderación de tejido de la publicación 103 de la *International Comision on Radiation Protection*^6^^,^^8^. En el CALDose X se introdujeron los parámetros técnicos y las magnitudes dosimétricas asociadas con las radiografías por analizar. Es necesario especificar el tipo de estructura anatómica, la proyección realizada y el tipo de filtro según el equipo. Posteriormente, se muestran los resultados de las dosis absorbidas en órganos y las dosis efectivas. Para las estimaciones mostradas en este trabajo, se introdujeron los valores promedio de estas magnitudes para cada equipo y tipo de radiografía. Los riesgos de incidencia y mortalidad por cáncer también son proporcionados por CALDose X. Para su estimación se considera el riesgo atribuible de por vida según el número de casos por cada 100.000 personas expuestas a una dosis de 100 mGy, de acuerdo con lo publicado por el *National Research Council*^9^.

Para medir la dosis de radiación dispersa recibida por los neonatos vecinos, se utilizó una cámara de ionización de marca Fluke, modelo Victoreen, dispuesta a un metro de la superficie del paciente en dirección horizontal, ya que esta es la distancia mínima a la cual se encuentran las incubadoras contiguas. Se obtuvo una tasa de dosis equivalente en unidades de μSv/h y se multiplicó por el tiempo de exposición (tiempo de disparo en horas). Para estimar la dosis efectiva en μSv, se estableció un factor de ponderación igual a uno para la calidad de la radiación usada. Además, a la distancia de medición y teniendo en cuenta las dimensiones de los pacientes, se puede considerar que la exposición de todo el cuerpo es uniforme.

Se realizó un estudio observacional de cohorte concurrente. Las variables cuantitativas se expresaron en términos de medias con desviación estándar o medianas con rango intercuartílico, según su distribución, mientras que las variables cualitativas se mostraron como frecuencias absolutas y relativas. Se usó el programa Stata para el análisis estadístico.

El protocolo de investigación fue aprobado por el Comité de Investigaciones y Ética en Investigaciones del Hospital Universitario San Ignacio.

## Resultados

En el estudio se incluyeron 160 pacientes entre abril y septiembre de 2021. En el cuadro 1, se describen las características generales de los neonatos que requirieron al menos una radiografía durante su estancia en la Unidad de Recién Nacidos del Hospital Universitario San Ignacio.

**Cuadro 1:** Características de los neonatos que requirieron radiografías durante su estancia en la Unidad de Recién Nacidos del Hospital Universitario San Ignacio (n = 160).

Variables cuantitativas		Media ± DE, mediana (RIC)
	Edad gestacional al nacer (semanas)	37 (34 - 38)
	Edad gestacional corregida al momento de tomar la radiografía (semanas)	37,2 (34 - 39,1)
	Edad cronológica al momento de toma de la radiografía (días)	5 (1 - 24)
	Peso al nacer (gramos)	2583 (2053 - 3123)
	Peso al momento de la toma de la radiografía (gramos)	2530 (1883 - 3110)
	Talla al nacer (cm)	46 (42,3 - 49)
	Talla al momento de la toma de la radiografía (cm)	46 (42 - 49)
	Edad materna (años)	28 ± 6,7
**Variables cualitativas**		**n (%)**
	Sexo masculino	87 (54,4)
	Nacimiento por cesárea	122 (76,3)
**Características de las radiografías tomadas**		
**Indicaciones de las radiografías**		**n (%)**
	Dificultad respiratoria	123 (24,8)
	Verificación de localización de catéteres centrales	59 (11,9)
	Intolerancia a la nutrición entérica	32 (6,5)
	Enterocolitis necrosante	27 (5,5)
	Cardiopatía congénita	10 (2)
	Malformaciones congénitas	6 (1,2)
	Otras indicaciones	322 (65,1)
	Ninguna	9 (1,8)
Tipo de radiografía		**n (%)**
	Radiografía de tórax	322 (65,4)
	Radiografía de abdomen	166 (33,7)
	Otras radiografías (clavícula, cráneo, etc.)	4 (0,8)
Número de radiografías		Mediana (RIC) (mínimo-máximo)
	Por paciente	2 (1,5) (1- 30)

DE: desviación estándar; RIC: rango intercuartílico

Se tomaron 492 radiografías en los 160 pacientes, con diferentes indicaciones para el estudio. Como principal indicación, se encontró dificultad respiratoria en 123 (24,9 %) neonatos, seguida de verificación de la localización de un catéter central en 59 (11,9 %) y, en tercer lugar, intolerancia a la nutrición entérica en 32 (6,5 %). En 9 (1,8 %) de ellos, no se encontró indicación para la toma de la radiografía. La mayoría de los pacientes fueron de sexo masculino (n = 87; 54,4 %) y la vía del parto más frecuente fue la cesárea (n = 122; 76,3 %).

La radiografía de tórax fue la que se tomó con mayor frecuencia (n=322; 65,4 %), seguida de la radiografía de abdomen (n=166; 33,7 %) y otras, como radiografías de clavículas, cráneo y extremidades (n=4; 0,8 %). La mediana para el número de radiografías por paciente fue de dos, con un mínimo de una radiografía por paciente y un máximo de 30 radiografías. En el cuadro 1, se describen las características de las radiografías tomadas.

El Hospital Universitario San Ignacio cuenta con dos equipos portátiles: un equipo en modalidad de radiografía computarizada y el digital directo. El mayor porcentaje de radiografías se tomó con el equipo computarizado (n=352; 71,5 %) y el resto con el digital directo (n=140; 28,4 %).

En el cuadro 2, se describe la dosis de entrada para el grupo poblacional y para cada equipo, el computarizado y el digital, respectivamente. Se encontró que la mediana de la dosis de entrada fue de 0,095 (0,022 - 0,126) mGy. La mediana de la dosis de entrada con el equipo computarizado fue de 0,112 mGy (rango: 0,022- 0,134) y con el equipo digital fue de 0,020 mGy (rango: 0,019-0,022).

**Cuadro 2:** Dosis de entrada en la superficie

Dosis de entrada en piel	Mediana (RIC)
Dosis de entrada general (mGy)	0,095 (0,022 - 0,126)
Dosis de entrada (mGy) equipo CR	0,112 (0,022 - 0,134)
Dosis de entrada (mGy) equipo DR	0,020 (0,019 - 0,022)

RIC: rango intercuartílico; CR: radiografía computarizada;

DR: radiografía digital

Los valores técnicos y las magnitudes dosimétricas relacionadas con la técnica usada para la toma de radiografías, y el cálculo de las dosis efectivas, se muestran en el cuadro 3. Se relacionan los valores promedio, la dosis de entrada efectiva y el producto dosis-área (obtenido sólo en el equipo digital).

**Cuadro 3:** Parámetros técnicos y magnitudes dosimétricas para calcular las dosis efectivas

Equipo	Examen	Corriente (V)	Potencia (mA)	Dosis de entrada en la superficie (mGy)	Producto dosis-área (Gy•cm^2^)
CR	Tórax AP	60	0,25	0,023	-------
	Abdomen AP	60	0,25	0,021	-------
DR	Tórax AP	60	1	0,108	0,004
	Abdomen AP	60	1,2	0,113	0,005

CR: radiografía computarizada; DR: radiografía digital; AP: antero-posterior; DPA: producto dosis-área

Los valores obtenidos de las dosis efectivas estimadas se presentan en el cuadro 4. Con el equipo digital se pueden obtener dos valores de dosis efectiva debido a que este cuenta con un medidor de producto dosis-área, ausente en el equipo computarizado. La comparación de los valores de dosis efectiva entre los dos equipos se debe hacer con base en la misma magnitud que, en este caso, es la dosis de entrada en la superficie.

**Cuadro 4:** Dosis efectivas estimadas

Equipo	Examen	Magnitud usada	Dosis efectiva (mSv)
CR	Tórax AP	Dosis de entrada en la superficie	0,019
	Abdomen AP	Dosis de entrada en la superficie	0,021
DR	Tórax AP	Dosis de entrada en la superficie	0,004
	Abdomen AP	Dosis de entrada en la superficie	0,004
	Tórax AP	Producto dosis-área	0,007
	Abdomen AP	Producto dosis-área	0,009

CR: radiografía computarizada; DR: radiografía digital; AP: antero-posterior

La dosis efectiva que reciben los pacientes por una radiografía de tórax es 4,75 veces mayor con el equipo computarizado que con el digital y, asimismo, por una radiografía de abdomen, es 5,25 veces mayor. Con el equipo digital, las dosis efectivas obtenidas con base en el producto dosis-área, son mayores a las obtenidas con la dosis de entrada en la superficie. Esto implicaría que calcular las dosis efectivas con base en la dosis de entrada en la superficie, hace subestimarlas con un factor de 2.

Lo anterior teniendo en cuenta que el producto dosis-área es un valor medido directamente, que caracteriza la salida del haz y el tamaño del campo utilizado, y además, que se verifica durante los controles de calidad del equipo, mientras que la estimación de la dosis de entrada en la superficie implica una incertidumbre mayor, por los cálculos necesarios y el ajuste del rendimiento.

Una vez calculadas las respectivas dosis efectivas, se determinaron las dosis absorbidas en los diferentes órganos, tanto para la radiografía de tórax como para la de de abdomen, y con los equipos computarizado y digital. También, se identificaron probabilidades de riesgo de incidencia y mortalidad por cáncer según el sexo y por cada grupo mencionado. En lo que respecta a la probabilidad de riesgo radiológico, en orden de relevancia, los órganos que presentan mayor riesgo con una radiografía de tórax son glándula mamaria, timo y pared cardíaca, y con una de abdomen, son hígado, colon y estómago. Se evidenció mayor probabilidad de riesgo de incidencia y de mortalidad por cáncer en los grupos cuyos estudios radiográficos se tomaron con el equipo computarizado, en comparación con el digital.

El promedio de la dosis de radiación dispersa para algunas de las radiografías fue de 4,17 (0,3-18,7). Se estimó que una dosis equivalente por radiación dispersa y, por ende, dosis efectiva por radiación dispersa, fue de 2,3x10^-7^ mSv. Este valor se considera poco significativo respecto a los valores de dosis efectivas por la toma de una radiografía, ya que es cinco veces menor que la dosis efectiva estimada.

Se realizó un análisis multivariado con regresión lineal múltiple. La variable dependiente fue la dosis de entrada generada por cada radiografía, y las variables independientes fueron edad cronológica, edad corregida al momento de la toma de la radiografía, sexo, peso al nacer, talla al nacer y tipo de equipo. Con este análisis, se pudo identificar que la toma de una radiografía con el equipo computarizado aumenta la dosis de entrada en la superficie en 0,174 mGy. Además, se puede considerar que la dosis depende más de la madurez del neonato, ejemplificada por la edad gestacional corregida al momento del estudio, que por otros factores adicionales. Este modelo fue el más parsimonioso y explicó la dosis de entrada en la superficie en un 53 %.

Posteriormente, se validaron los coeficientes para cada variable independiente del modelo, con la prueba estadística t de Student. Se encontró que las variables tipo de equipo y edad gestacional corregida, tienen una relación lineal con la dosis de entrada en la superficie final. La interacción entre la edad gestacional corregida y el uso del equipo computarizado fue estadísticamente significativa (p=0,019). Por cada semana que disminuye la edad gestacional corregida, se evidencia un aumento de la dosis de entrada en la superficie a razón de 0,0024 mGy con el equipo computarizado (cuadro 5).

**Cuadro 5:** Resumen del modelo

Modelo	Número de radiografías	R^2^	R^2^ ajustado			Error estándar de la estimación
1	492	0,5315	0,5287			0,1707
		**Coeficientes no estandarizados**				**Intervalo de confianza**
**Modelo**						
		**B**	**Error estándar**	**t**	**P > (t)**	**95 %**
1	(Constante)	0,008	0,0335	0,24	0,810	-0,5771 a 0,0738
	Tipo de equipo 1	0,1741	0,3737	4,66	0,000	0,1007 a 0,2475
	EGC	0,0003	0,0009	0,42	0,676	-0,001 a 0,0021
	Interacción	-0,0024	0,0010	-2,36	0,019	-0,0043 a -0,0004

* Variable dependiente: dosis de entrada en piel; EGC: edad gestacional corregida

## Discusión

El uso diagnóstico de radiografías en la práctica clínica se sigue presentando con igual o mayor frecuencia a nivel mundial, pero se subestiman los riesgos que conlleva, en especial, en las unidades de cuidado neonatal. Esta práctica genera un riesgo mayor por exposición a la radiación que en los adultos debido a: mayor expectativa de vida, mayor tiempo de vulnerabilidad por mayor proliferación y diferenciación celular en esta etapa, y mayor impacto del haz de rayos X sobre los tejidos por su pequeño tamaño ^1^^-^^4^.

A nivel mundial, en los estudios y descripciones se identifican como principales factores de riesgo en pacientes que se exponen a radiación: edad cronológica, edad gestacional corregida, peso al nacer y al momento de la toma de radiografía, talla al nacer y al tomar la radiografía, así como valores técnicos dosimétricos al usar los equipos de radiología. Sin embargo, cabe anotar que estos estudios no incluyen las variables mencionadas en la cuantificación de las dosis mediante datos estadísticos por regresión lineal múltiple ^10^^-^^13^.

Con las variables analizadas, se encontró correlación entre los resultados y las características generales de la población neonatal, como gran frecuencia de enfermedades respiratorias, especialmente en neonatos de sexo masculino nacidos por cesárea, siendo la radiografía de tórax la más solicitada.

En la población de estudio, los principales factores que incidieron en el aumento de dosis de radiación fueron la edad gestacional corregida y el uso del equipo computarizado.

El mayor porcentaje de radiografías se tomó con el equipo computarizado (n=352; 71,5 %), en comparación con el equipo digital (n=140; 28,4 %), debido a que por protocolo institucional el equipo digital está destinado a pacientes con aislamientos microbiológicos específicos y casos sospechosos o confirmados de infección por SARS-CoV-2, condiciones menos frecuentes en los neonatos.

La dosis de entrada en la superficie fue de 0,112 mGy (0,022-0,134 mGy) con el equipo computarizado y de 0,020 mGy (0,019-0,02 mGy) con el equipo digital. Se obtuvo una dosis de entrada en la superficie 5,6 veces mayor con el equipo computarizado, ya que la tecnología del dispositivo digital permite que la radiografía tenga mejor calidad con menores valores de carga y, por ende, menor dosis de entrada.

Se detectó que en el 1,8 % de los casos no se tenía indicación específica para la toma de la radiografía. Con la comparación entre las dosis aportadas por estos dos tipos de equipos, fue evidente el aumento de la dosis de radiación al utilizar el equipo computarizado, así, cobra relevancia el concepto expuesto por la Sociedad Latinoamericana de Radiología Pediátrica, de que todas las exposiciones a la radiación deben mantenerse tan bajas como sea razonablemente posible (*As Low As Reasonably Achievable*, ALARA). Por lo tanto, estos hallazgos pueden ser una oportunidad para reconocer la importancia de optimizar los registros clínicos que justifiquen las indicaciones e interpretación de los estudios, con el fin de disminuir la toma de radiografías innecesarias e, incluso, modificar la elección del estudio por otro que genere menos radiación ^2^^,^^14^^,^^15^.

En un estudio en México se encontró que es posible reducir la dosis de radiación en más del 40 % en una población neonatal, sin que esto tenga ningún efecto sobre la calidad de las radiografías de tórax. Esto es posible mediante la modificación de la técnica convencional (aumento de kV y disminución de mAS) y con la adición de un filtro adicional. La consideración de esta posibilidad es relevante para mejorar la calidad en futuras atenciones ^11^.

Es importante mencionar que el Hospital Universitario San Ignacio cuenta con procesos rigurosos de control de calidad para cada equipo que son aplicados anualmente. La Resolución 482 de 2018 establece un control bianual para garantizar el buen desempeño de los equipos ^16^.

En la literatura científica regional actual, hay un estudio publicado por la Universidad de Antioquia, en el cual no se encontraron diferencias significativas en las dosis efectivas calculadas para el equipo computarizado. Sin embargo, con el análisis de las variables adicionales evaluadas en el presente trabajo (peso, talla, edad gestacional, tipo de parto, tipo de radiografía, tipo de equipo empleado), se demostró que la interacción entre menores edades gestacionales corregidas y el uso de un equipo computarizado influye significativamente en el aumento de la dosis efectiva. Asimismo, se estimaron las dosis de entrada, de dispersión, absorbidas y efectivas, y se identificaron factores que influyen directamente sobre el aumento de estas dosis ^17^.

Reconociendo el impacto que tienen los estudios radiológicos en la población neonatal, se propone idear investigaciones de seguimiento que permitan dilucidar el impacto a largo plazo de la radiación en pacientes con mayor número de estudios radiológicos y conformar estrategias de chequeo, para sensibilizar a todos los profesionales implicados sobre la importancia del proceso. Además, sería útil realizar estudios similares en cada institución para evaluar las dosis y los riesgos, de acuerdo con las características de la población y el tipo de equipo disponible.

Las dosis de radiación recibidas por los neonatos para la toma radiográfica con un equipo de tecnología computarizada, es mayor en comparación con las radiografías tomadas con tecnología digital. Por esta razón, las instituciones que cuentan con estos dos tipos de tecnología deben adoptar medidas para hacer uso de la tecnología digital en la toma de radiografías en las unidades de cuidado neonatales.
